# Leptomeningeal Cells Transduce Peripheral Macrophages Inflammatory Signal to Microglia in Reponse to *Porphyromonas gingivalis* LPS

**DOI:** 10.1155/2013/407562

**Published:** 2013-12-02

**Authors:** Yicong Liu, Zhou Wu, Xinwen Zhang, Junjun Ni, Weixian Yu, Yanmin Zhou, Hiroshi Nakanishi

**Affiliations:** ^1^Department of Aging Science and Pharmacology, Faculty of Dental Science, Kyushu University, Fukuoka 812-8582, Japan; ^2^Department of Implantology, School of Stomatology, Jilin University, Changchun 130021, China

## Abstract

We report here that the leptomeningeal cells transduce inflammatory signals from peripheral macrophages to brain-resident microglia in response to *Porphyromonas gingivalis (P.g.)* LPS. The expression of Toll-like receptor 2 (TLR2), TLR4, TNF-**α**, and inducible NO synthase was mainly detected in the gingival macrophages of chronic periodontitis patients. In *in vitro* studies, *P.g.* LPS induced the secretion of TNF-**α** and IL-1**β** from THP-1 human monocyte-like cell line and RAW264.7 mouse macrophages. Surprisingly, the mean mRNA levels of TNF-**α** and IL-1**β** in leptomeningeal cells after treatment with the conditioned medium from *P.g.* LPS-stimulated RAW264.7 macrophages were significantly higher than those after treatment with *P.g.* LPS alone. Furthermore, the mean mRNA levels of TNF-**α** and IL-1**β** in microglia after treatment with the conditioned medium from *P.g.* LPS-stimulated leptomeningeal cells were significantly higher than those after *P.g.* LPS alone. These observations suggest that leptomeninges serve as an important route for transducing inflammatory signals from macrophages to microglia by secretion of proinflammatory mediators during chronic periodontitis. Moreover, propolis significantly reduced the *P.g.* LPS-induced TNF-**α** and IL-1 **β** production by leptomeningeal cells through inhibiting the nuclear factor-**κ**B signaling pathway. Together with the inhibitory effect on microglial activation, propolis may be beneficial in preventing neuroinflammation during chronic periodontitis.

## 1. Introduction

Periodontitis is the most common adult chronic inflammatory disorder, which results in a consequence of the persistent systemic inflammatory responses [1, 2]. *Porphyromonas gingivalis *(*P.g.*) is the major *periodontopathic* bacteria [3, 4], and its LPS (*P.g.* LPS) is thought to induce periodontitis through Toll-like receptors, TLR2 or TLR4 [5]. As the main population in inflammatory oral mucosa, macrophages are known to determine *P.g.* LPS-induced oral innate immune responses through TLRs during chronic periodontitis [5, 6]. Macrophages can be polarized into M1 and M2 phenotypes depending on the microenvironment [7]. M1 macrophages promote inflammation and tissue damage by secreting proinflammatory mediators, including TNF-*α*, IL-1*β*, and expressing inducible NO synthase (iNOS). In contrast, M2 macrophages promote anti-inflammation and wound healing by secreting anti-inflammatory mediators, including IL-10 and TGF-*β*1, and upregulating arginase 1 (Arg 1) [8, 9]. In addition to causing chronic systemic inflammatory diseases, including atherosclerosis, cardiovascular disease, and diabetes [10–12], periodontitis has been proposed as a risk factor for the central nervous system (CNS) disorders, such as Alzheimer's disease (AD) [13–15]. However, the exact route by which periodontitis transduces the peripheral inflammatory messages into the CNS remains unclear.

Besides the physical role as the cerebrospinal fluid-blood barrier [16, 17], the leptomeninges also play roles as secretory cells, which transduce systemic inflammatory signals into the CNS [18–20]. Furthermore, TLR2 and TLR4 are detected in cultured human leptomeningeal cell lines [21] and leptomeninges of experimental animals [22, 23], suggesting that leptomeninges are involved in the innate response of the CNS. Moreover, increasing evidence shows that microglia are the primary brain cells that respond to systemic inflammatory stimuli to play their well-known roles in neuroinflammation [24–27].

Propolis is a resinous substance produced by honeybees as a defense against intruders. It has relevant therapeutic properties that have been used since ancient times. The chemical composition of propolis depends on the local flora at the site of collection [28, 29]. Considering its antioxidative and anti-inflammatory effects [30–32], propolis may have protective effects against neuroinflammatory responses.

In the present study, we have attempted to examine possible roles of leptomeninges in transducing inflammatory signals from peripheral macrophages to brain-resident microglia in response to *P.g.* LPS stimulation. The mean amounts of TNF-*α* and IL-1*β* secreted by leptomeningeal cells after treatment with the conditioned medium from *P.g.* LPS-stimulated macrophages were significantly higher than those after treatment with *P.g.* LPS alone. Furthermore, the mean amounts of TNF-*α* and IL-1*β* secreted by microglia after treatment with the conditioned medium from *P.g.* LPS-treated leptomeningeal cells were significantly higher than those after treatment with *P.g.* LPS alone. These observations suggest that leptomeninges transduce inflammatory signals from peripheral macrophages to brain-resident microglia by secreting inflammatory mediators during chronic periodontitis. Moreover, propolis significantly reduced the *P.g.* LPS-induced TNF-*α* and IL-1*β* production by leptomeningeal cells through inhibiting the nuclear factor-*κ*B (NF-*κ*B) signaling pathway. Together with our recent findings of direct inhibitory effects on the microglial inflammatory responses, propolis may be beneficial in preventing neuroinflammation during chronic periodontitis.

## 2. Materials and Methods

### 2.1. Reagents

*P.g.* LPS were purchased from InvivoGen (San Diego, CA, USA). Propolis was purchased from Yamada Bee Farm Corporation (Okayama, Japan), function blocking antibodies to TLR2, TLR4 and isotype control antibodies were purchased from eBioscience (San Diego, CA, USA). Bay 11-7082, a specific NF-*κ*B inhibitor, was purchased from Sigma-Aldrich (ST. Louis MO, USA). Antibodies of mouse anti-TLR2 (T2.5), mouse anti-TLR4 (HTA-125) were purchased from eBioscience (San Diego, CA, USA). Mouse anti-phospho-I*κ*B*α*, rabbit anti-I*κ*B*α*, goat anti-TNF-*α*, and rabbit anti-actin were purchased from Santa Cruz Biotechnology (Delaware Avenue Santa Cruz, CA). Mouse anti-iNOS (4E5) was purchased from Abcam (Heidelberg, Germany). Rabbit anti-ionized calcium binding adaptor molecule (Iba1) was purchased from Wako Chem Industries Ltd (Osaka, Japan).

### 2.2. Tissue Preparation from Periodontitis Patients

The gingival samples were obtained from patients undergoing periodontal surgery or extraction. The periodontal diagnosis of subjects with chronic periodontitis was established based on clinical and radiographic criteria defined by the 1999 International World Workshop for a Classification of Periodontal Diseases and Conditions [33]. The samples included 9 cases diagnosed as chronic periodontitis (aged 34–60, 6 males and 3 females), which were recruited from Periodontology Department of School of Stomatology, Jilin University. Following surgery, excised gingival specimens were immediately placed in liquid nitrogen and subsequently frozen at −80°C until the following experiments.

Gingival samples were immersed in the periodate lysine paraformaldehyde (PLP) fixative consisting of 0.01 M sodium metaperiodate, 0.075 M L-lysine-HCl, 4% paraformaldehyde, and 0.03% phosphate buffer (pH 6.2) for 6 h at 4°C. The specimens were cryoprotected 2 days in 30% sucrose in phosphate-buffered saline and then were embedded in an optimal cutting temperature compound (Sakura Finetechnical Co., Ltd., Tokyo, Japan). Serial coronal frozen sections (14 *μ*m) were subjected to the immunohistochemical analyses [34, 35].

### 2.3. Double-Immunofluorescent Staining

The sections were hydrated and treated with 10% donkey serum for 1 h at 24°C and then were incubated with each primary antibody overnight at 4°C. The primary mouse monoclonal anti-TLR2 (T2.5, 1 : 200), mouse monoclonal anti-TLR4 (HTA-125, 1 : 200), goat polyclonal anti-TNF-*α* (1 : 200), and mouse monoclonal anti-iNOS (4E5, 1 : 500) antibodies were mixed with rabbit polyclonal anti-Iba1 (1 : 500) antibody. After washing with PBS, the sections were incubated with a mixture of FITC-conjugated and rhodamine-conjugated secondary antibodies for 2 h at 24°C. After washing, the sections were mounted in the antifading medium Vectashield (Vector Laboratory) and examined by a confocal laser scanning microscope (CLSM) LSM510MET (CLSM, C2si, Nikon, Japan). CLSM images of individual sections were taken as a stack at 1 *μ*m step size along *z*-direction with 20 × objectives (Numerical Aperture = 0.5), zoom factor 1.0. A rectangle (1024 × 1024 pixels) corresponding to the size of 450 × 450 *μ*m was used as the counting frame. CLSM images were shown as the middle of the stacked images.

### 2.4. THP-1 Human Monocyte-Like Cell Line and RAW264.7 Mouse Macrophage Culture

THP-1 cells which were purchased from the ATCC (Manassas, VA, USA) were cultured in RPMI-1640 medium supplemented with 10% fetal bovine serum (FBS, ICN Biomedicals, Eschwege, Germany), 0.05 mM 2-mercaptoethanol, penicillin G (40 U/mL), and streptomycin (50 *μ*g/mL). RAW264.7 cells which were purchased from the ATCC were cultured in Minimum Essential Medium Alpha (MEM-*α*, GIBCO, USA) supplemented with 10% FBS, penicillin G (40 U/mL), and streptomycin (50 *μ*g/mL); those cells were cultured at 37°C in a humidified atmosphere with 5% CO_2_.

### 2.5. Leptomeningeal Cell Culture

Leptomeningeal cells were prepared from the brain of 3-day-old C57black/6N mouse. Dissected leptomeningeal tissues were plated on poly-D-lysine-coated culture dishes (one mouse/mm^2^) and incubated in Dulbecco's Modified Eagle's medium (DMEM, Nissui Pharmaceutical co., Ltd., Japan) containing 10% FBS, penicillin G (40 U/mL), and streptomycin (50 *μ*g/mL) at 37°C in a humidified atmosphere with 5% CO_2_ for 7 days. At this time, any contaminated cells such as neuronal and glial cells were removed by shaking and were washed twice with Ca^2+^/Mg^2+^-free sterile isotonic buffer, pH 7.0, which consisted of 137 mM NaCl, 5 mM KCl, 0.7 mM KH_2_PO_4_, 25 mM glucose, 59 mM sucrose, 0.3% bovine serum albumin, penicillin (40 U/mL), and streptomycin (50 *μ*g/mL). The purity of the leptomeningeal cells was more than 96% as determined by the immunostaining of fibronectin [18, 19].

### 2.6. Microglial Cell Culture

The c-myc-immortalized mouse microglial cell line, MG6 (RIKEN Cell Bank, Tsukuba, Japan), was maintained in DMEM containing 10% FBS supplemented with 100 *μ*M *α*-mercaptoethanol, 10 *μ*g/mL insulin, 100 *μ*g/mL streptomycin, and 100 U/mL penicillin (BD Falcon, Franklin Lakes, NJ) [36, 37].

### 2.7. Real-Time Quantitative RT-PCR Analysis

THP-1 and RAW264.7 cells were treated with *P.g.* LPS (1 *μ*g/mL) for 24 h, leptomeningeal cells were incubated with the conditioned medium from *P.g.* LPS- or *P.g.* LPS-treated RAW264.7 cells (MCM) for 4 h, and MG6 were incubated with the conditioned medium from *P.g.* LPS- or *P.g.* LPS-treated leptomeningeal cells (LCM) for 24 h. The mRNA isolated from *P.g.* LPS-treated or nontreated cells were subjected to real-time quantitative RT-PCR. The total RNA was extracted with the Purelink RNA microkit (Invitrogen, Japan) according to the manufacturer's instructions. A total of 800 ng of extracted RNA was reverse transcribed to cDNA using the High Capacity RNA-to-cDNA Master Mix (Applied Biosystems, Foster City, CA). The thermal cycling was held at 50°C for 2 min, and then at 95°C for 10 min, followed by 40 cycles of 95°C for 15 s and 60°C for 1 min. The cDNA was amplified in duplicate using TaqMan Universal PCR Master Mix (Applied Biosystems, Foster City, CA) with an Applied Biosystems 7500/7500 Fast Real-Time PCR System. The data were evaluated using the 7500 software program (version 2.0, Applied Biosystems). The primer sequences used were as follows: iNOS: 5′-GCC ACC AAC AAT GGC AAC A-3′ and 5′-CGT ACC GGA TGA GCT GTG AAT T-3′; Arginase-1: 5′-CGC CTT TCT CAA AAG GAC AG-3′ and 5′-CCA GCT CTT CAT TGG CTT TC-3′; TNF-*α*: 5′-ATG GCC TCC CTC TCA GTT C-3′and 5′-TTG GTG GTT TGC TAC GAC GTG-3′; IL-1*β*: 5′-CAA CCA ACA AGT GAT ATT CTC CAT G-3′ and 5′-GAT CCA CAC TCT CCA GCT GCA-3′; IL-10: 5′-ATG CTG CCT GCT CTT ACT GAC TG-3′ and 5′-CCC AAG TAA CCC TTA AAG TCC TGC-3′. For data normalization, an endogenous control (actin) was assessed to control for the cDNA input, and the relative units were calculated by a comparative Ct method. All real-time RT-PCR experiments were repeated three times, and the results are presented as the means of the ratios ± SEM.

### 2.8. ELISA Assay

THP-1 and RAW264.7 cells were treated with *P.g.* LPS (1 *μ*g/mL), leptomeningeal cells were treated with *P.g.* LPS (100 ng/mL), and the condition medium was collected at 6 h, 24 h, 48 h, and 72 h after *P.g.* LPS treatment. RAW264.7 were incubated with propolis (15 *μ*g/mL) 1 h before *P.g.* LPS treatment, and the condition medium was collected at 48 h after treatment. The leptomeningeal cells were incubated with propolis (10 *μ*g/mL) 1 h before *P.g.* LPS treatment, and the condition medium was collected at 6 h after treatment. In the separated experiments, RAW264.7, leptomeningeal cells, and MG6 were treated with TLR2 (10 *μ*g/mL), TLR4 (10 *μ*g/mL) antibodies or the control antibodies or Bay 11-7082 (20 *μ*M) 1 h before *P.g.* LPS treatment. The condition medium was collected at the time points after the reagents treatment. TNF-*α* and IL-1*β* released from THP-1, RAW264.7, leptomeningeal cells, and MG6 were measured using enzyme-linked immunosorbent assay (ELISA) kits (R&D Systems) following the protocol provided by the manufacturer. The absorbency at 450 nm was measured using a microplate reader.

### 2.9. Determination of Cell Viability

RAW264.7 and leptomeningeal cells were seeded in 96-well plates for 24 h (5 × 10^3^ cells/well) then incubated with various concentrations of propolis for 48 h. Cell viability was assessed using the Cell-Counting Kit-8 (CCK-8) (Dojindo, Kumamoto, Japan) according to the manufacturer's instructions. Briefly, after propolis treatment, 10 *μ*L CCK-8 was added to each well and incubated at 37°C for 2 h. The optical density was read at a wavelength of 450 nm with a microplate reader. Cell viability was calculated using the following formula: optical density of treated group/control group × 100%.

### 2.10. Electrophoresis and Immunoblotting

RAW264.7 and leptomeningeal cells were cultured at a density of 5 × 10^5^ cells/mL, and the cytosolic samples of RAW264.7 and leptomeningeal cells were collected at 30 min, 60 min, and 120 min after *P.g.* LPS (1 *μ*g/mL, 100 ng/mL) treatment with or without propolis (15 *μ*g/mL, 10 *μ*g/mL). The samples were electrophoresed in 12% SDS-polyacrylamide gels, and the proteins on SDS gels were transferred electrophoretically to nitrocellulose membranes. Following the blocking, the membranes were incubated at 4°C overnight under gentle agitation with each primary antibody: rabbit anti-I*κ*B*α* (1 : 1000), mouse anti-pI*κ*B*α* (1 : 1000) antibodies. After washing, the membranes were incubated with horseradish peroxidase (HRP-) labeled anti-rabbit (1 : 2000, GE Healthcare, UK) or anti-mouse (1 : 2000, GE Healthcare, UK) antibodies for 2 h at 24°C, then the protein bands were detected by an enhanced chemiluminescence detection system (ECK kit, Amersham Pharmacia Biotech) using an image analyzer (LAS-4000, Fuji Photo Film, Tokyo, Japan).

### 2.11. Data Analysis

The data are represented as the means ± SEM. The statistical analyses were performed using a one-way or two-way analysis of variance (ANOVA) with a post hoc Tukey's test using the GraphPad Prism software package. A value of *P* < 0.05 was considered to indicate statistical significance (GraphPad Software Inc., San Diego, CA, USA).

## 3. Results

### 3.1. Characterization of Macrophages in Human Gingival Tissues of Periodontitis and Cultured Macrophages after *P.g.* LPS Stimulation

**Figure 1:**
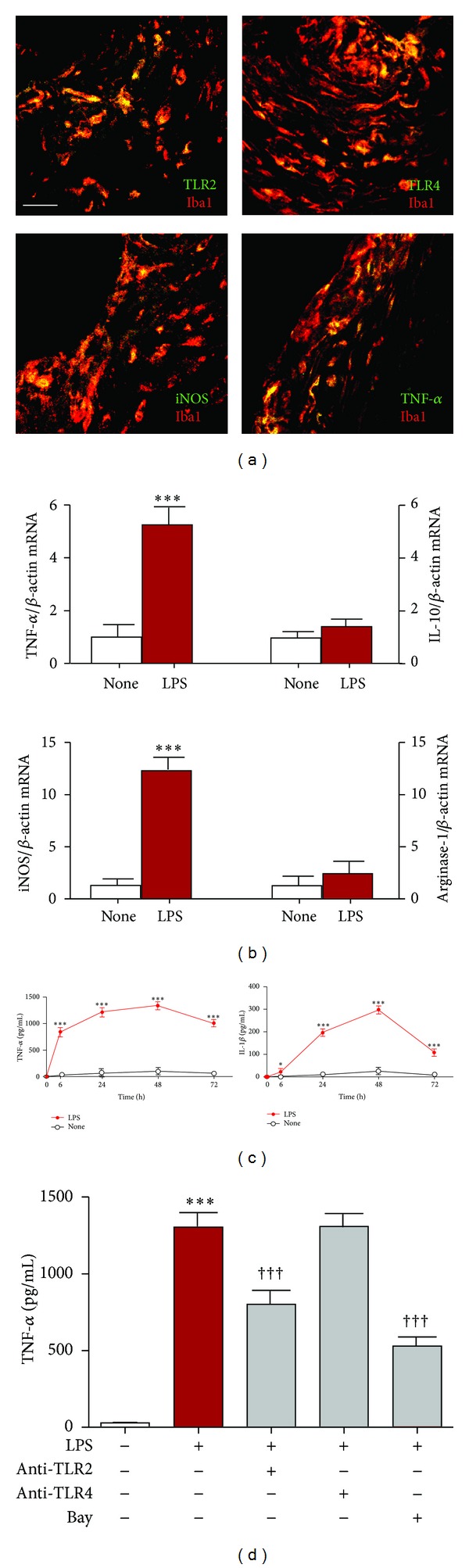
Characterization of macrophages in gingiva of chronic periodontitis patients and cultured macrophages after *P.g.* LPS stimulation. Immunofluorescent CLMS images of TLR2, TLR4, TNF-*α*, and iNOS in gingiva of chronic periodontitis patients (a), scale bar = 20 *μ*m. The mean mRNA levels of TNF-*α*, IL-10, iNOS, and Arg I of THP-1 human monocyte-like cell line after *P.g.* LPS (LPS, 1 *μ*g/mL) treatment for 24 h (b). Data are presented by mean ± SEM (*n* = 3), ***P < 0.001 versus nontreated cells (none). Time course of TNF-*α* and IL-1*β* release in RAW264.7 mouse macrophages after *P.g.* LPS treatment (c). Data are presented by mean ± SEM (*n* = 4, each), ***P < 0.001, *P < 0.05 versus nontreated cells (none). The LPS-induced TNF-*α* secretion from RAW264.7 mouse macrophages at 48 h with the neutralizing antibodies against TLR2 (10 *μ*g/mL), TLR4 (10 *μ*g/mL), or a specific NF-*κ*B inhibitor, Bay 11-7082 (Bay, 20 *μ*M) (d). Data are presented by mean ± SEM (*n* = 4, each), ***P < 0.001 versus nontreated cells (none), and ^†††^ *P* < 0.001 versus *P.g.* LPS alone.

We first examined the localization of TLR2, TLR4, and cytokines in human gingival tissues of chronic periodontitis patients, because macrophages are the main population in gingival tissues of chronic periodontitis to response *P.g.* LPS through TLR2 and TLR4 [6, 38]. Our immunofluorescent double staining revealed that the immunoreactivities for TLR2, TLR4, TNF-*α*, and iNOS corresponded well with those for Iba1 (Figure 1(a)) and their correspondence ratios were 72%, 79%, 53%, and 65%, respectively. However, immunoreactivities for IL-10 and TGF-*β*1 were rarely found in human periodontitis gingival tissues (data not shown). We further determined the macrophage phenotypes after treatment with *P.g.* LPS using THP-1 human monocyte-like cell line and RAW264.7 mouse macrophages. In comparison to the nontreated cells, both the mean mRNA expression levels of TNF-*α* and iNOS were significantly increased in THP-1 cells after treatment with *P.g.* LPS (1 *μ*g/mL). However, mean mRNA expression levels of IL-10 and Arg 1 were not significantly increased after treatment with *P.g.* LPS (Figure 1(b)). The similar results were also obtained in RAW264.7 cells (data not shown). Furthermore, the time-dependent release of TNF-*α* and IL-1*β* from RAW264.7 cells was induced from 6 h and peaked at 48 h and then was decreased gradually later after *P.g.* LPS treatment (Figure 1(c)). The similar results were also obtained in THP-1 cells (data not shown). Moreover, *P.g.* LPS-induced TNF-*α* and IL-1*β* production in RAW264.7 cells was significantly suppressed by anti-TLR2 antibody, but not by anti-TLR4 antibody (Figure 1(d)). On the other hand, the control antibodies with the same concentration had no significant effect (data not shown). These observations confirm that macrophages are polarized to M1 phenotype in response to *P.g.* LPS stimulation through TLR2.

### 3.2. Secretion of Proinflammatory Mediators by Leptomeningeal Cells after Treatment with the Conditioned Medium from *P.g.* LPS-Treated Macrophages and *P.g.* LPS

**Figure 2:**
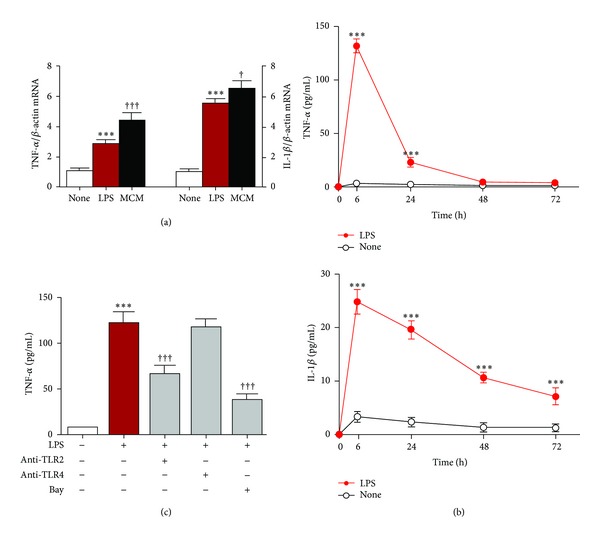
Secretion of proinflammatory mediators by leptomeningeal cell after treatment with the conditioned medium from *P.g.* LPS-treated macrophages and *P.g.* LPS. The mean mRNA levels of TNF-*α* and IL-1*β* of leptomeningeal cells at 4 h incubated with *P.g.* LPS (LPS) and conditioned medium of *P.g.* LPS-treated RAW264.7 mouse macrophages (MCM) (a). Data are presented by mean ± SEM (*n* = 3), ***P < 0.001 versus nontreated cells (none). ^†††^ *P* < 0.001 versus *P.g.* LPS alone. Time course of TNF-*α* and IL-1*β* secreted by leptomeningeal cells after treatment with *P.g.* LPS (100 ng/mL) (b). Data are presented by mean ± SEM (*n* = 4, each), ***P < 0.001 versus nontreated cells (none). The *P.g.* LPS-induced TNF-*α* secretion by leptomeningeal cells at 6 h with the neutralizing antibodies against TLR2 (10 *μ*g/mL), TLR4 (10 *μ*g/mL), or Bay 11-7082 (Bay, 20 *μ*M) (c). Data are presented by mean ± SEM (*n* = 4, each), ***P < 0.001 versus nontreated cells (none), and ^†††^ *P* < 0.001 versus *P.g.* LPS alone.

We next used mouse primary cultured leptomeningeal cells to address whether they could respond to inflammatory mediators secreted from *P.g.* LPS-treated macrophages using MCM and *P.g.* LPS alone. Surprisingly, the mean expression levels of TNF-*α* and IL-1*β* mRNA in leptomeningeal cells were significantly increased from 4 h after treatment with MCM in comparison to those observed after treatment with *P.g.* LPS alone (Figure 2(a)). Furthermore, in comparison to the nontreated cells, the secretion of TNF-*α* and IL-1*β* from leptomeningeal cells peaked at 6 h, decreased quickly, and it is noted that TNF-*α* was undetected at 48 h after treatment with *P.g.* LPS (Figure 2(b)). Moreover, secretion of TNF-*α* from leptomeningeal cells after treatment with *P.g.* LPS was significantly suppressed by anti-TLR2 antibody, but not by anti-TLR4 antibody (Figure 2(c)). On the other hand, the control antibodies with the same concentration had no significant effect (data not shown). To date, these observations provide the first evidence that leptomeningeal cells are polarized to proinflammatory phenotype in response to inflammatory signals from *P.g.* LPS-induced macrophages through TLR2.

### 3.3. Secretion of Proinflammatory Mediators by Microglia after Treatment with the Conditioned Medium from *P.g.* LPS-Treated Leptomeningeal Cells and *P.g.* LPS

**Figure 3:**
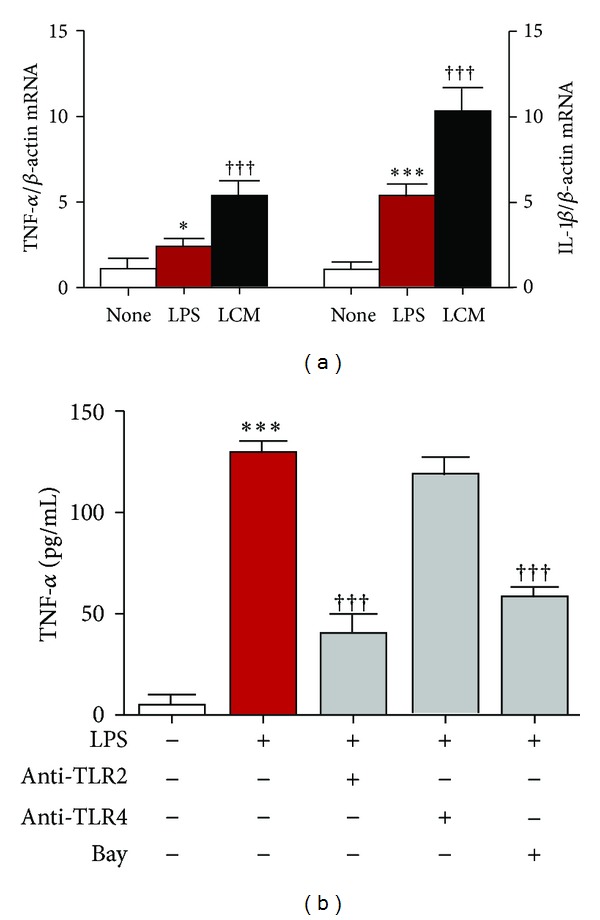
Secretion of proinflammatory mediators by microglia after treatment with the conditioned medium from *P.g.* LPS-treated leptomeningeal cells and *P.g.* LPS. The mean mRNA levels of TNF-*α* and IL-1*β* of MG6 microglia at 24 h incubated with *P.g.* LPS (LPS) and the conditioned medium of leptomeningeal cells (LCM) after treatment with *P.g.* LPS (a). Data are presented by mean ± SEM (*n* = 3), *P < 0.05, ***P < 0.001 versus nontreated cells (none). ^†††^ *P* < 0.001 versus *P.g.* LPS alone. The *P.g.* LPS-induced TNF-*α* secretion in MG6 microglia at 48 h with the neutralizing antibodies against TLR2 (10 *μ*g/mL), TLR4 (10 *μ*g/mL), or Bay 11-7082 (Bay, 20 *μ*M) (b). Data are presented by mean ± SEM (*n* = 4, each), ***P < 0.001 versus nontreated cells (none), and ^†††^ *P* < 0.001 versus *P.g.* LPS alone.

We have previously demonstrated that the leptomeninges are involved in the cytokine production by glial cells during chronic systemic inflammation [18–20]. In order to confirm that the leptomeninges could trigger microglial inflammatory responses, we next examined the mRNA expression of TNF-*α* and IL-1*β* in MG6 microglia after treatment with the conditioned medium from *P.g.* LPS-treated leptomeningeal cells (LCM). The mean mRNA expression levels of TNF-*α* and IL-1*β* were significantly increased after 24 h treatment with LCM in comparison to those observed after treatment with *P.g.* LPS alone (Figure 3(a)). We further examined the microglial responses after treatment with *P.g.* LPS, because *P.g.* LPS has been recently found in AD brain [15]. The mean level of TNF-*α* secreted by microglia was significantly increased from 6 h and peaked at 48 h after treatment with *P.g.* LPS (100 ng/mL) in comparison to that by nontreated microglia. The amount of IL-1*β* secreted from microglia also reached peak at 48 h after treatment with *P.g.* LPS (data not shown). The *P.g.* LPS-induced secretion of TNF-*α* by microglia was significantly suppressed by anti-TLR2 antibody, but not by anti-TLR4 antibody (Figure 3(b)). However, the control antibodies with the same concentration had no significant effect (data not shown). These observations clearly demonstrate that microglia were polarized to proinflammatory M1-like phenotype in response to inflammatory signals from *P.g.* LPS-induced leptomeningeal cells through TLR2.

### 3.4. Effect of Propolis on *P.g.* LPS-Induced Proinflammatory Phenotypes of Macrophages and Leptomeningeal Cells

**Figure 4:**
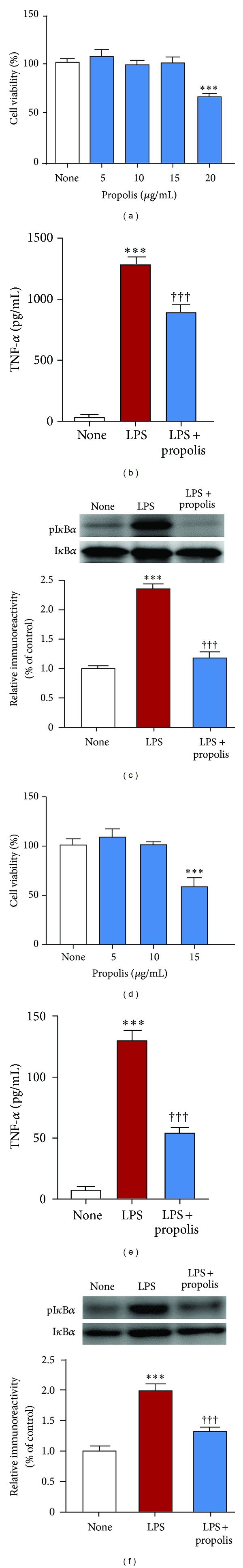
Effect of propolis on *P.g.* LPS-induced proinflammatory phenotypes of macrophages and leptomeningeal cells. Cell viability in RAW264.7 mouse macrophages (a) and in leptomeningeal cells (d) in the absence and presence of propolis with different concentrations for 48 h. Data are presented by mean ± SEM (*n* = 3), ***P < 0.001 versus nontreated cells (none). TNF-*α* release from *P.g.* LPS-treated RAW264.7 mouse macrophages with or without propolis (15 *μ*g/mL) for 48 h (b), and leptomeningeal cells with or without propolis (10 *μ*g/mL) for 6 h (e) were measured by ELISA. Data are presented by mean ± SEM (*n* = 4, each), ***P < 0.001 versus nontreated cells (none), and ^†††^ *P* < 0.001 versus *P.g.* LPS alone. Phosphorylation of I*κ*B*α* and the quantitative analyses of immunoblots in RAW264.7 mouse macrophages (c) and leptomeningeal cells (f) at 30 min after expose to *P.g.* LPS with or without propolis. Data are presented by mean ± SEM (*n* = 4, each), ***P < 0.001 versus nontreated cells (none), and ^†††^ *P* < 0.001 versus *P.g.* LPS alone.

Finally, the effects of propolis on the secretion of *P.g.* LPS-induced proinflammatory mediators by macrophages and leptomeningeal cells were examined, because propolis has antioxidative and anti-inflammatory effects [31, 32]. The mean cell viability was not significantly changed with the final concentrations until 15 *μ*g/mL on RAW264.7 macrophages (Figure 4(a)) and the final concentrations until 10 *μ*g/mL on leptomeningeal cells after treatment with propolis (Figure 4(d)). Therefore, we used propolis with the concentration of 15 *μ*g/mL on RAW264.7 and 10 *μ*g/mL on leptomeningeal cells, respectively, for the following experiments. In comparison to the nontreated cells, pretreatment with propolis significantly inhibited TNF-*α* secretion by macrophages (Figure 4(b)) and leptomeningeal cells (Figure 4(e)) after treatment with *P.g.* LPS. The effects of propolis on the *P.g.* LPS-induced NF-*κ*B activation were then examined, because NF-*κ*B regulates the expression of proinflammatory mediators, including TNF-*α* and IL-1*β*. The expression of I*κ*B*α* phosphorylation was significantly increased from 30 min in both RAW264.7 and leptomeningeal cells after treatment with *P.g.* LPS. Pre-treatment with propolis significantly inhibited the *P.g.* LPS-induced phosphorylation of I*κ*B*α* in RAW264.7 cells (Figure 4(c)) and leptomeningeal cells (Figure 4(f)). These observations demonstrate that propolis suppresses the *P.g.* LPS-induced proinflammatory responses by inhibiting the NF-*κ*B signaling pathway in both peripheral macrophages and leptomeningeal cells.

## 4. Discussion

The major findings of the present study are that leptomeninges transduce *P.g.* LPS-induced inflammatory signals from peripheral macrophages to brain-resident microglia, resulting in the induction of neuroinflammation. Furthermore, propolis was found to attenuate the secretion of *P.g.* LPS-induced proinflammatory mediators by leptomeningeal cells. To date, this is the first report to highlight that the leptomeninges serve as an important route for transducing peripheral inflammatory signals to the CNS during chronic periodontitis.

As the main population in inflammatory oral mucosa, macrophages phenotypes are known to determine *P.g.* LPS-induced oral innate immune responses through TLRs during periodontitis [5]. In the present study, we confirmed that macrophages densely expressed TLR2, TLR4, TNF-*α*, and iNOS in the gingival tissues of chronic periodontitis patients. However, proinflammatory M1 macrophages are not limited to the infected gingiva but also increased in the circulation during chronic periodontitis [39, 40]. The present observations indicate that *P.g.* LPS stimulation significantly increased the mean levels of mRNA expression of TNF-*α* and iNOS, but not those of IL-10 and Arg1, suggesting that macrophages are polarized to M1 phenotype in response to *P.g.* LPS during chronic periodontitis. Furthermore, the mean amounts of *P.g.* LPS-induced TNF-*α* and IL-1*β* secreted by macrophages were significantly suppressed by anti-TLR2 antibody, but not by anti-TLR4 antibody, further indicating that macrophages respond to *P.g.* LPS mainly through TLR2 [38, 41, 42], but not through TLR4 [5, 42].

Systemic inflammation and infections could worsen a number of CNS disorders [26, 43]. Among the common chronic inflammatory disorders in adults, much attention has been paid to the periodontitis as the pathogenesis of CNS disorders, including AD [13, 15, 44]. The increase in macrophage-derived TNF-*α* and IL-1*β* in the circulation during periodontitis [39, 40] also supports the idea that chronic periodontitis is involved in the pathogenesis of systemic inflammatory diseases, including atherosclerosis, cardiovascular disease, and diabetes [10–12]. Therefore, it is reasonable to consider that the increased M1 polarization of macrophages during chronic periodontitis can be also a risk factor for AD, because the elevated levels of TNF-*α* and IL1-*β* are associated not only with the cognitive decline but also with the progression of AD [45–47]. Recently, we have reported that leptomeninges provide a critical link between chronic systemic inflammation and subsequent neuroinflammation [18–20]. In the present study, we have found that *P.g.* LPS stimulates both THP-1 and RAW264.7 macrophages to secrete TNF-*α* and IL-1*β*. Furthermore, the mean mRNA expression levels of TNF-*α* and IL-1*β* in leptomenigeal cells were significantly increased as early as 4 h after treatment with MCM. These observations indicate that leptomenigeal cells could respond to proinflammatory mediators secreted from M1 macrophages during chronic periodontitis. Furthermore, *P.g.* LPS also stimulates leptomeningeal cells to produce TNF-*α* and IL-1*β*, which is consistent with previous studies using E coli LPS [18, 19] and other meningitis causable agents [48–50]. Importantly, unlike peripheral macrophages, leptomeningeal cells produce TNF-*α* and IL-1*β* from 6 h after treatment with *P.g.* LPS. Considering the inhibitory effect of TLR2 antibody on *P.g.* LPS-induced production of proinflammatory mediators, leptomeningeal cells could respond to *P.g.* LPS more sensitively than peripheral macrophages through TLR2, but not through TLR4, even though TLR4 is also expressed in meningeal cells [21, 23].

Microglia are well-known key players of neuroinflammation [26, 27], which are activated by TNF-*α* and IL-1*β* in an autocrine manner [51, 52]. The present findings indicate that LCM significantly enhanced the mRNA expression of TNF-*α* and IL-1*β* in microglia, suggesting that proinflammatory mediators secreted from *P.g.* LPS-treated leptomenigeal cells could subsequently activate microglia to generate neuroiflammation during chronic periodontitis. Furthermore, our present observations demonstrate that the mean levels of TNF-*α* and IL-1*β* secreted by microglia were significantly increased after treatment with *P.g.* LPS alone. Furthermore, *P.g.* LPS-induced secretion of proinflammatory mediators by microglia was significantly suppressed by anti-TLR2 antibody. These observations support the recent idea that *P.g.* LPS may be involved in the progression of AD [15]. Furthermore, TLR2 is increased in peripheral blood mononuclear cells from AD patients [53]. Therefore, it is reasonable to consider that leptomenigeal cells may transduce the periodontitis-derived inflammatory signals to microglia, resulting in powerful neuroninflammatory responses. Further investigations will be necessary to examine a possible involvement of other glial cells during chronic periodontitis, because other glial cells, such as astrocytes, also contribute to the CNS disorders [54, 55]. Moreover, further investigations are necessary to clarify the involvement of other factors in neuroinflammation during chronic periodontitis, because *P.g.* LPS is only one of the related factors of chronic periodontitis.

Neuroprotective drug therapies have not yet translated well from the lab to the clinic because of an excessive focus of treatments on promoting the survival of neurons, with far less work on nonneuronal brain cells. Recently, leptomeninges have been focused on delivering compounds/genes to brain [56, 57]. Therefore, meninges can be considered as the direct targets for treating CNS disorders, including AD. Propolis is a resinous substance produced by honeybees as a defense against intruders. It has relevant therapeutic properties that have been used since ancient times. Depending on their antioxidative and anti-inflammatory effects [30–32], we here provide the first evidence that propolis can significantly inhibit TNF-*α* and IL-1*β* production by both RAW264.7 cells and leptomenigeal cells through inhibiting the NF-*κ*B signaling pathway, because NF-*κ*B is a critical transcription factor that encodes genes of proinflammatory mediators, including TNF-*α* and IL-1*β* [58]. These findings agreed well with our previous observations that propolis significantly inhibited hypoxia-induced NF-*κ*B-dependent production of proinflammatory mediators by microglia. Recently, we have reported that the exaggerated neuroinflammatory responses evoked by microglia are responsible for an impairment of the hippocampal long-term potentiation in the middle-aged animals subjected to adjuvant arthritis [59]. Therefore, the efficient attenuation of propolis in *P.g.* LPS-induced NF-*κ*B-dependent proinflammatory pathway of leptomeningeal cells and microglia may prevent the age-dependent exaggerated neuroinflammatory responses in the CNS.

## 5. Conclusion

In conclusion, our present findings strongly suggest that the leptomeninges serve as an important route for transducing inflammatory signals from peripheral macrophages into brain-resident microglia by secreting proinflammatory mediators during chronic periodontitis. Propolis may benefit for preventing and reducing neuroinflammation in CNS disorders, including AD, by attenuating *P.g.* LPS-induced inflammatory signals from peripheral macrophages, leptomeningeal cells and microglia during chronic periodontitis. Further investigations are necessary to clarify the involvement of other factors in neuroinflammation during chronic periodontitis.

## References

[1] Eke PI, Dye BA, Wei L (2012). Prevalence of periodontitis in adults in the United States: 2009 and 2010. *Journal of Dental Research*.

[2] Rethman MP (2010). Inflammation in chronic periodontitis and significant systemic diseases. *Journal of the California Dental Association*.

[3] Seymour GJ, Ford PJ, Cullinan MP, Leishman S, Yamazaki K (2007). Relationship between periodontal infections and systemic disease. *Clinical Microbiology and Infection*.

[4] Yoshimura A, Hara Y, Kaneko T, Kato I (1997). Secretion of IL-1*β*, TNF-*α*, IL-8 and IL-1*α* by human polymorphonuclear leukocytes in response to lipopolysaccharides from periodontopathic bacteria. *Journal of Periodontal Research*.

[5] Darveau RP, Pham TT, Lemley K (2004). *Porphyromonas gingivalis* lipopolysaccharide contains multiple lipid A species that functionally interact with both toll-like receptors 2 and 4. *Infection and Immunity*.

[6] Foey AD, Crean S (2013). Macrophage subset sensitivity to endotoxin tolerisation by *Porphyromonas gingivalis*. *PLoS One*.

[7] Liu G, Yang H (2013). Modulation of macrophage activation and programming in immunity. *Journal of Cellular Physiology*.

[8] Gordon S (2003). Alternative activation of macrophages. *Nature Reviews Immunology*.

[9] Gordon S, Taylor PR (2005). Monocyte and macrophage heterogeneity. *Nature Reviews Immunology*.

[10] Hettne KM, Weeber M, Laine ML (2007). Automatic mining of the literature to generate new hypotheses for the possible link between periodontitis and atherosclerosis: lipopolysaccharide as a case study. *Journal of Clinical Periodontology*.

[11] Monteiro AM, Jardini MAN, Alves S (2009). Cardiovascular disease parameters in periodontitis. *Journal of Periodontology*.

[12] Pischon N, Heng N, Bernimoulin JP, Kleber BM, Willich SN, Pischon T (2007). Obesity, inflammation, and periodontal disease. *Journal of Dental Research*.

[13] Kamer AR, Craig RG, Dasanayake AP, Brys M, Glodzik-Sobanska L, de Leon MJ (2008). Inflammation and Alzheimer’s disease: possible role of periodontal diseases. *Alzheimer’s and Dementia*.

[14] Stein SP, Steffen MJ, Smith C (2012). Serum antibodies to periodontal pathogens are a risk factor for Alzheimer’s disease. *Alzheimer’s and Dementia*.

[15] Poole S, Singhrao SK, Kesavalu L, Curtis MA, Crean S (2013). Determining the presence of periodontopathic virulence factors in short-term postmortem Alzheimer's disease brain tissue. *Journal of Alzheimer’s Disease*.

[16] Nilsson C, Lindvall-Axelsson M, Owman C (1992). Neuroendocrine regulatory mechanisms in the choroid plexus-cerebrospinal fluid system. *Brain Research Reviews*.

[17] Tanno H, Nockels RP, Pitts LH, Noble LJ (1993). Immunolocalization of heat shock protein after fluid percussive brain injury and relationship to breakdown of the blood-brain barrier. *Journal of Cerebral Blood Flow and Metabolism*.

[18] Wu Z, Zhang J, Nakanishi H (2005). Leptomeningeal cells activate microglia and astrocytes to induce IL-10 production by releasing pro-inflammatory cytokines during systemic inflammation. *Journal of Neuroimmunology*.

[19] Wu Z, Hayashi Y, Zhang J, Nakanishi H (2007). Involvement of prostaglandin E2 released from leptomeningeal cells in increased expression of transforming growth factor-*β* in glial cells and cortical neurons during systemic inflammation. *Journal of Neuroscience Research*.

[20] Wu Z, Tokuda Y, Zhang XW, Nakanishi H (2008). Age-dependent responses of glial cells and leptomeninges during systemic inflammation. *Neurobiology of Disease*.

[21] Humphries HE, Triantafilou M, Makepeace BL, Heckels JE, Triantafilou K, Christodoulides M (2005). Activation of human meningeal cells is modulated by lipopolysaccharide (LPS) and non-LPS components of *Neisseria meningitidis* and is independent of Toll-like receptor (TLR)4 and TLR2 signalling. *Cellular Microbiology*.

[22] Laflamme N, Soucy G, Rivest S (2001). Circulating cell wall components derived from gram-negative, not gram-positive, bacteria cause a profound induction of the gene-encoding Toll-like receptor 2 in the CNS. *Journal of Neurochemistry*.

[23] Laflamme N, Rivest S (2001). Toll-like receptor 4: the missing link of the cerebral innate immune response triggered by circulating gram-negative bacterial cell wall components. *The FASEB Journal*.

[24] Perry VH, Newman TA, Cunningham C (2003). The impact of systemic infection on the progression of neurodegenerative disease. *Nature Reviews*.

[25] Perry VH (2004). The influence of systemic inflammation on inflammation in the brain: implications for chronic neurodegenerative disease. *Brain, Behavior, and Immunity*.

[26] Perry VH, Nicoll JAR, Holmes C (2010). Microglia in neurodegenerative disease. *Nature Reviews*.

[27] Akiyama H, Arai T, Kondo H, Tanno E, Haga C, Ikeda K (2000). Cell mediators of inflammation in the Alzheimer disease brain. *Alzheimer Disease and Associated Disorders*.

[28] Bankova VS, De Castro SL, Marcucci MC (2000). Propolis: recent advances in chemistry and plant origin. *Apidologie*.

[29] Marcucci M (1995). Propolis: chemical composition, biological properties and therapeutic activity. *Apidologie*.

[30] Banskota AH, Tezuka Y, Kadota S (2001). Recent progress in pharmacological research of propolis. *Phytotherapy Research*.

[31] Sforcin JM (2007). Propolis and the immune system: a review. *Journal of Ethnopharmacology*.

[32] Ramos AFN, De Miranda JL (2007). Propolis: a review of its anti-inflammatory and healing actions. *Journal of Venomous Animals and Toxins Including Tropical Diseases*.

[33] Armitage GC (1999). Development of a classification system for periodontal diseases and conditions. *Annals of Periodontology*.

[34] Wu Z, Nagata K, Iijima T (2000). Immunohistochemical study of NGF and its receptors in the synovial membrane of the ankle joint of adjuvant-induced arthritic rats. *Histochemistry and Cell Biology*.

[35] Wu Z, Nagata K, Iijima T (2002). Involvement of sensory nerves and immune cells in osteophyte formation in the ankle joint of adjuvant arthritic rats. *Histochemistry and Cell Biology*.

[36] Takenouchi T, Ogihara K, Sato M, Kitani H (2005). Inhibitory effects of U73122 and U73343 on Ca^2+^ influx and pore formation induced by the activation of P2X_7_ nucleotide receptors in mouse microglial cell line. *Biochimica et Biophysica Acta*.

[37] Nakamichi K, Saiki M, Kitani H (2006). Suppressive effect of simvastatin on interferon-*β*-induced expression of CC chemokine ligand 5 in microglia. *Neuroscience Letters*.

[38] Barksby HE, Nile CJ, Jaedicke KM, Taylor JJ, Preshaw PM (2009). Differential expression of immunoregulatory genes in monocytes in response to *Porphyromonas gingivalis* and *Escherichia coli* lipopolysaccharide. *Clinical and Experimental Immunology*.

[39] Golub LM, Payne JB, Reinhardt RA, Nieman G (2006). Can systemic diseases co-induce (not just exacerbate) periodontitis? A hypothetical “two-hit” model. *Journal of Dental Research*.

[40] Berker E, Kantarci A, Hasturk H, Van Dyke TE (2012). Blocking pro-inflammatory cytokine release modulates peripheral blood mononuclear cell response to *Porphyromonas gingivalis*. *Journal of Periodontology*.

[41] Zhang D, Chen L, Li S, Gu Z, Yan J (2008). Lipopolysaccharide (LPS) of *Porphyromonas gingivalis* induces IL-1*β*, TNF-*α* and IL-6 production by THP-1 cells in a way different from that of *Escherichia coli* LPS. *Innate Immunity*.

[42] Zhou Q, Desta T, Fenton M, Graves DT, Amar S (2005). Cytokine profiling of macrophages exposed to *Porphyromonas gingivalis*, its lipopolysaccharide, or its FimA protein. *Infection and Immunity*.

[43] Lei L, Li H, Yan F, Li Y, Xiao Y (2011). *Porphyromonas gingivalis* lipopolysaccharide alters atherosclerotic-related gene expression in oxidized low-density-lipoprotein-induced macrophages and foam cells. *Journal of Periodontal Research*.

[44] Noble JM, Borrell LN, Papapanou PN, Elkind MSV, Scarmeas N, Wright CB (2009). Periodontitis is associated with cognitive impairment among older adults: analysis of NHANES-III. *Journal of Neurology, Neurosurgery and Psychiatry*.

[45] Higuchi M, Hatta K, Honma T (2010). Association between altered systemic inflammatory interleukin-1*β* and natural killer cell activity and subsequently agitation in patients with Alzheimer disease. *International Journal of Geriatric Psychiatry*.

[46] Holmes C, Cunningham C, Zotova E (2009). Systemic inflammation and disease progression in Alzheimer disease. *Neurology*.

[47] Holmes C, Cunningham C, Zotova E, Culliford D, Perry VH (2011). Proinflammatory cytokines, sickness behavior, and Alzheimer disease. *Neurology*.

[48] Brandenburg LO, Varoga D, Nicolaeva N (2009). Expression and regulation of antimicrobial peptide rCRAMP after bacterial infection in primary rat meningeal cells. *Journal of Neuroimmunology*.

[49] Christodoulides M, Makepeace BL, Partridge KA (2002). Interaction of *Neisseria meningitidis* with human meningeal cells induces the secretion of a distinct group of chemotactic, proinflammatory, and growth-factor cytokines. *Infection and Immunity*.

[50] Fowler MI, Ho Wang Yin KY, Humphries HE, Heckels JE, Christodoulides M (2006). Comparison of the inflammatory responses of human meningeal cells following challenge with *Neisseria lactamica* and with *Neisseria meningitidis*. *Infection and Immunity*.

[51] Sheng WS, Hu S, Kravitz FH, Peterson PK, Chao CC (1995). Tumor necrosis factor alpha upregulates human microglial cell production of interleukin-10 *in vitro*. *Clinical and Diagnostic Laboratory Immunology*.

[52] Lee YB, Nagai A, Kim SU (2002). Cytokines, chemokines, and cytokine receptors in human microglia. *Journal of Neuroscience Research*.

[53] Zhang W, Wang LZ, Yu JT, Chi ZF, Tan L (2012). Increased expressions of TLR2 and TLR4 on peripheral blood mononuclear cells from patients with Alzheimer’s disease. *Journal of the Neurological Sciences*.

[54] Verkhratsky A, Olabarria M, Noristani HN, Yeh C-Y, Rodriguez JJ (2010). Astrocytes in Alzheimer’s disease. *Neurotherapeutics*.

[55] Steele ML, Robinson SR (2012). Reactive astrocytes give neurons less support: implications for Alzheimer’s disease. *Neurobiology of Aging*.

[56] Madhavan D, Mirowski P, Ludvig N (2008). Effects of subdural application of lidocaine in patients with focal epilepsy. *Epilepsy Research*.

[57] Ludvig N, Switzer RC, Tang HM, Kuzniecky RI (2012). Autoradiographic evidence for the transmeningeal diffusion of muscimol into the neocortex in rats. *Brain Research*.

[58] Liu SF, Malik AB (2006). NF-*κ*B activation as a pathological mechanism of septic shock and inflammation. *American Journal of Physiology—Lung Cellular and Molecular Physiology*.

[59] Liu X, Wu Z, Hayashi Y, Nakanishi H (2012). Age-dependent neuroinflammatory responses and deficits in long-term potentiation in the hippocampus during systemic inflammation. *Neuroscience*.

